# Supplementary material for: Building and experimenting with an agent-based model to study the population-level impact of CommunityRx, a clinic-based community resource referral intervention
Source: PLoS Comput Biol. 2021 Oct 25;17(10):e1009471. doi: 10.1371/journal.pcbi.1009471 (PMC8568099; doi:10.1371/journal.pcbi.1009471)

**Table A. Agent-Based Model Parameters, Description, Values and Sources of Values**

| **Parameter** | **Parameter Description** | **Notation** | **Values or Range** | **Source(s) of values** |
| --- | --- | --- | --- | --- |
| alpha | Activation level | $\alpha$ | $\in(0,1)$ | Derived from Skolasky et al 2011^1^ |
| gamma | Inertia unique to a service / resource | $\gamma$ | $\left\{ \begin{aligned} L=0.1 \\ M=0.3 \\ H=0.75 \end{aligned} \right.$ | Relative values based on survey of expert informants^a^ |
| delta | Distance threshold for a resource | $\delta$ | $\left\{ \begin{aligned} L=0.05 \\ M=0.1 \\ H=0.15 \end{aligned} \right.$ | Derived using data from a population-based study self-reported use of community resources by resource type; straight line distance from home to resource used was calculated and categorized by tertiles by resource type^2^ |
| decay (dosing) | Rate of receding knowledge level (of a resource) | $\lambda$ | $\in(0.991,\ldots$  $,0.9994)$ | Chosen in reference to dynamic β values to exhibit temporal decay, which had face validity when presented to subject matter experts and are further supported by prior work^3,4^ |
| dosing effect | dosing value for a resource, based on the source of the information | $\epsilon_{x}$ | $\left\{ \begin{aligned} doctor=0.05 \\ nurse=0.15 \\ psr=0.25 \\ use=0.2 \\ peer=0.9 \end{aligned} \right.$ | Relative values informed by expert informants^a^ and through sensitivity analyses |
| p-score | Propensity of an agent to receive information based on activity. | $propensity$ | $\left\{ \begin{aligned} none=0.0001 \\ L=0.005 \\ M=0.025 \\ H=0.05 \end{aligned} \right.$ | Values derived from an iterative close-coded survey administered to expert informants (N=14),^a^ who reported likelihood (low, medium, high) that an agent would receive resource information from another agent while doing a given activity. |
| H, high; L, low; M, medium; PSR, patient service representative.  ^a^Expert informant include a diversity of researchers (N=14) from a variety of disciplines (medicine, computer science, bioinformatics, epidemiology) with expertise about the geographic region and its population. | | | | |

**References**

1. Skolasky RL, Green AF, Scharfstein D, Boult C, Reider L, Wegener ST. Psychometric properties of the patient activation measure among multimorbid older adults. Health Serv Res. 2011;46: 457–478. doi:10.1111/j.1475-6773.2010.01210.x
2. Garibay L, Makelarski JA, Lindau ST. South Side Population Health Study. 2014 pp. 1–9. Available: <https://thestudies.uchicago.edu/sites/sshvs.uchicago.edu/files/uploads/The%20South%20Side%20Population%20Health%20Study%20Report.pdf>
3. Candia C, Jara-Figueroa C, Rodriguez-Sickert C, Barabasi A, Hidalgo C. The universal decay of collective memory and attention. Nature Human Behavior. 2019;3: 82-91.
4. Kaligotla C, Ozik J, Collier N, Macal CM, Boyd K, Makelarski J, et al. Model Exploration of an Information-Based Healthcare Intervention Using Parallelization and Active Learning. JASSS. 2020;23(4): 1.

|  | n | % |
| --- | --- | --- |
| Sex |  |  |
| Male | 351,633 | 43.8 |
| Female | 450,558 | 56.2 |
| Race |  |  |
| White | 197,871 | 24.5 |
| African American or Black | 473,017 | 59.0 |
| Asian | 15,720 | 2.0 |
| Other or more than 1 race | 115,583 | 14.4 |
| Ethnicity |  |  |
| Hispanic | 131,731 | 16.4 |
| Non-Hispanic | 670,460 | 83.6 |
| Age |  |  |
| 16-24 years | 139,617 | 17.4 |
| 25-44 years | 282,993 | 34.3 |
| 45-64 years | 253,694 | 31.6 |
| 65 or more years | 125,887 | 15.7 |

**Table B. Demographic Characteristics of the Synthetic Population (N=802,191) Generated Using Data From the Synthetic Populations and Ecosystems of the World; Chicago, IL 2016-2018.**

**Appendix A: Summary Model Description following the Overview, Design concepts and Details (ODD) Protocol (Grimm, 2020)**

A complete, detailed model description that covers all elements of the ODD protocol (Grimm, 2020) is provided in Kaligotla et al. (2018). The overall purpose of the CRx ABM is to:

1. demonstrate the flow and spread of resource info from primary agents (those who received the CommunityRx (CRx) informational intervention via a HealtheRx) to others in the community and
2. conduct experiments on how delivery of the HealtheRx and other variable conditions can impact the flow and spread of information to the community.

Specifically, we are addressing the following questions:

1. Can we recreate, *in silico*, delivery of the CRx intervention, including generation of personalized HealtheRxs (“clinical dosing”)?
2. Can we recreate the underlying dynamics of resource information diffusion (“social dosing”) following *in silico* delivery of an intervention delivered to primary agents at the point of clinical care?
3. How does variation in clinical parameters (e.g., who delivers the HealtheRx to the primary agent) affect information diffusion to the broader population?

To consider our model realistic enough for its purpose, we used the following criteria:

1. the demographic characteristics of the synthetic population would reflect those of the actual population (within a select geographical area (in our case, a 16 ZIP code area in Chicago),
2. the synthetic population would exhibit diverse behavioral characteristics, including agent activity and knowledge evolution patterns,
3. *in silico* delivery of the intervention would be replicated with high fidelity,
4. large-scale information diffusion networks would emerge among agents exchanging resource information,
5. varying the propensity for information exchange would result in networks with different topological characteristics.

The model includes the following entities: a synthetic population of agents and a geographical environment that together are statistically representative of the community under study (16 ZIP codes on Chicago’s South Side, a predominantly African American/Black demographic where the CRx intervention was created and studied). The state variables characterizing these entities are listed in Table A in S1 Text. As for the spatial and temporal resolution and extent: Each time step in the model represents one hour of simulated time and simulations are run for a period of at least 4 weeks. Agents are assigned specific activity schedules for every 24 hours, representing a simulated day. The model is spatially explicit, with each place in the synthetic environment representing an actual physical place with a fixed latitude and longitude in the 16 ZIP code study region. The extent of the CRx ABM is in recreating the CRx intervention within the defined spatial and temporal resolution.

The most important processes of the model, which are repeated at every time step, are:

1. Assignment of a daily activity schedule for each agent in the model.

For each simulated hour of a day, every agent selects the activity for that hour. Agents then select a known place that corresponds to that activity (e.g., work at assigned workplaces, home care at home, medical care at clinics).

1. Agents are initialized with and maintain a β knowledge score for up to 200 resources that are updated at every time step. This β score is dynamic based on dosing events (receiving an intervention) as well as agent activity and interactions with other agents.
2. Agents co-locate in a place where they share information about their known places (social dosing). Information sharing (dosing) is dependent on the propensity to share information, which is based on the amenability of the activity itself to information sharing (e.g., the activity “sleeping” has a propensity score of none).
3. Agents’ decision to use a resource is modeled as a binary choice A/B Decision Model. Each activity is characterized by a decision type – A/B decision or not. Activities classified as health maintenance or promotion activities (the activities that the CRx intervention targets) face an A/B decision choice. Other activities not related to health-maintenance behaviors do not require a decision choice – agents proceed with such activity at the designated location. Agents presented with an ATUS activity in their daily schedule that is subject to an A/B decision face a choice to use a resource or not. The choice of using a resource dictates the location of the agent for each simulated hour.

The most important design concepts of the model are: (1) the A/B decision model, where agents choose whether to do a health-promoting activity or not, (2) the dynamics of information diffusion, where each agent’s knowledge about resources evolves based on the dosing source (clinical or social) and resource use after every time step of the simulation, and (3) *in silico* delivery of the personalized HealtheRx

**Table C. Common parameter values and ranges for direct versus social dosing experiment runs.**

Selected list of CommunityRx ABM model parameters with values and ranges that are varied in simulation runs. This table as well as the details of the CommunityRx ABM model, along with a comprehensive parameter descriptions, values and ranges have been previously published (Kaligotla, 2018; Kaligotla, 2020).

| MODEL PARAMETERS | BRIEF DESCRIPTION | VALUES / RANGE |
| --- | --- | --- |
| Dosing.Decay | Rate of knowledge attrition | (0.9910 to 0.9994) in increments of (0.0006) |
| Dosing.Peer | Dosing from network peer (social dosing) | (0.8 to 0.95) in increments of (0.1) |
| Gamma.Med | Resource inertia of performing a moderate activity | (1 to 3) in increments of (0.143) |
| Propensity.Multiplier | Multiplier applied to propensity of information sharing | (0.5 to 1.5) in increments of (0.0714) |
| Delta.Multiplier | Multiplier applied to distance threshold | (0.5 to 1.5) in increments of (0.0714) |

**Table D. Varied parameter settings experiment runs.**

Parameter settings for 45 simulation runs on the CommunityRx ABM. Each model run simulated the intervention for 4 weeks and model exhibited stable behavior by week 3. Model output from week 3 was used to characterize model parameter space. Experiment consisted of 15 runs for each HealtheRx information delivery source (doctor, nurse, and PSR) and designed to capture stochastic variation across different parameter settings.

| RUN | RANDOM.SEED | DOSING.DECAY | DOSING.PEER | GAMMA.MED | PROPENSITY.MULTIPLIER | DELTA.MULTIPLIER | HRX.SOURCE |
| --- | --- | --- | --- | --- | --- | --- | --- |
| 1 | 1 | 0.993857 | 0.907143 | 1.142857 | 1.285714 | 1.357143 | Doctor |
| 2 | 1 | 0.993857 | 0.907143 | 1.142857 | 1.285714 | 1.357143 | Nurse |
| 3 | 1 | 0.993857 | 0.907143 | 1.142857 | 1.285714 | 1.357143 | PSR |
| 4 | 2 | 0.993857 | 0.907143 | 1.142857 | 1.285714 | 1.357143 | Doctor |
| 5 | 2 | 0.993857 | 0.907143 | 1.142857 | 1.285714 | 1.357143 | Nurse |
| 6 | 2 | 0.993857 | 0.907143 | 1.142857 | 1.285714 | 1.357143 | PSR |
| 7 | 3 | 0.993857 | 0.907143 | 1.142857 | 1.285714 | 1.357143 | Doctor |
| 8 | 3 | 0.993857 | 0.907143 | 1.142857 | 1.285714 | 1.357143 | Nurse |
| 9 | 3 | 0.993857 | 0.907143 | 1.142857 | 1.285714 | 1.357143 | PSR |
| 10 | 1 | 0.993286 | 0.885714 | 1.428571 | 1.214286 | 1.071429 | Doctor |
| 11 | 1 | 0.993286 | 0.885714 | 1.428571 | 1.214286 | 1.071429 | Nurse |
| 12 | 1 | 0.993286 | 0.885714 | 1.428571 | 1.214286 | 1.071429 | PSR |
| 13 | 2 | 0.993286 | 0.885714 | 1.428571 | 1.214286 | 1.071429 | Doctor |
| 14 | 2 | 0.993286 | 0.885714 | 1.428571 | 1.214286 | 1.071429 | Nurse |
| 15 | 2 | 0.993286 | 0.885714 | 1.428571 | 1.214286 | 1.071429 | PSR |
| 16 | 3 | 0.993286 | 0.885714 | 1.428571 | 1.214286 | 1.071429 | Doctor |
| 17 | 3 | 0.993286 | 0.885714 | 1.428571 | 1.214286 | 1.071429 | Nurse |
| 18 | 3 | 0.993286 | 0.885714 | 1.428571 | 1.214286 | 1.071429 | PSR |
| 19 | 1 | 0.994429 | 0.896429 | 1.285714 | 1.142857 | 1.214286 | Doctor |
| 20 | 1 | 0.994429 | 0.896429 | 1.285714 | 1.142857 | 1.214286 | Nurse |
| 21 | 1 | 0.994429 | 0.896429 | 1.285714 | 1.142857 | 1.214286 | PSR |
| 22 | 2 | 0.994429 | 0.896429 | 1.285714 | 1.142857 | 1.214286 | Doctor |
| 23 | 2 | 0.994429 | 0.896429 | 1.285714 | 1.142857 | 1.214286 | Nurse |
| 24 | 2 | 0.994429 | 0.896429 | 1.285714 | 1.142857 | 1.214286 | PSR |
| 25 | 3 | 0.994429 | 0.896429 | 1.285714 | 1.142857 | 1.214286 | Doctor |
| 26 | 3 | 0.994429 | 0.896429 | 1.285714 | 1.142857 | 1.214286 | Nurse |
| 27 | 3 | 0.994429 | 0.896429 | 1.285714 | 1.142857 | 1.214286 | PSR |
| 28 | 1 | 0.991571 | 0.864286 | 1.285714 | 1 | 1.142857 | Doctor |
| 29 | 1 | 0.991571 | 0.864286 | 1.285714 | 1 | 1.142857 | Nurse |
| 30 | 1 | 0.991571 | 0.864286 | 1.285714 | 1 | 1.142857 | PSR |
| 31 | 2 | 0.991571 | 0.864286 | 1.285714 | 1 | 1.142857 | Doctor |
| 32 | 2 | 0.991571 | 0.864286 | 1.285714 | 1 | 1.142857 | Nurse |
| 33 | 2 | 0.991571 | 0.864286 | 1.285714 | 1 | 1.142857 | PSR |
| 34 | 3 | 0.991571 | 0.864286 | 1.285714 | 1 | 1.142857 | Doctor |
| 35 | 3 | 0.991571 | 0.864286 | 1.285714 | 1 | 1.142857 | Nurse |
| 36 | 3 | 0.991571 | 0.864286 | 1.285714 | 1 | 1.142857 | PSR |
| 37 | 1 | 0.992714 | 0.896429 | 1 | 0.857143 | 1.5 | Doctor |
| 38 | 1 | 0.992714 | 0.896429 | 1 | 0.857143 | 1.5 | Nurse |
| 39 | 1 | 0.992714 | 0.896429 | 1 | 0.857143 | 1.5 | PSR |
| 40 | 2 | 0.992714 | 0.896429 | 1 | 0.857143 | 1.5 | Doctor |
| 41 | 2 | 0.992714 | 0.896429 | 1 | 0.857143 | 1.5 | Nurse |
| 42 | 2 | 0.992714 | 0.896429 | 1 | 0.857143 | 1.5 | PSR |
| 43 | 3 | 0.992714 | 0.896429 | 1 | 0.857143 | 1.5 | Doctor |
| 44 | 3 | 0.992714 | 0.896429 | 1 | 0.857143 | 1.5 | Nurse |
| 45 | 3 | 0.992714 | 0.896429 | 1 | 0.857143 | 1.5 | PSR |

**Table E. Direct versus Social Dosing Experiment Results**

Results from 45 simulation runs on the CommunityRx ABM. Multiplier is the ration of number of agents retaining information only from social dosing to agents retaining information from clinical dosing.

| RUN | # AGENTS RETAINING INFORMATION FROM CLINICAL DOSING | # AGENTS RETAINING INFORMATION ONLY FROM SOCIAL DOSING | MULTIPLIER - SOCIAL DOSING / CLINICAL DOSING |
| --- | --- | --- | --- |
| 1 | 27392 | 126018 | 4.6 |
| 2 | 28880 | 124417 | 4.3 |
| 3 | 29812 | 124382 | 4.2 |
| 4 | 27458 | 123556 | 4.5 |
| 5 | 29147 | 124409 | 4.3 |
| 6 | 30128 | 122728 | 4.1 |
| 7 | 27530 | 124966 | 4.5 |
| 8 | 29242 | 124235 | 4.2 |
| 9 | 29912 | 124218 | 4.2 |
| 10 | 27797 | 125973 | 4.5 |
| 11 | 28833 | 123688 | 4.3 |
| 12 | 29681 | 122227 | 4.1 |
| 13 | 27622 | 123412 | 4.5 |
| 14 | 29283 | 124567 | 4.3 |
| 15 | 30052 | 122923 | 4.1 |
| 16 | 27962 | 125231 | 4.5 |
| 17 | 29206 | 124768 | 4.3 |
| 18 | 29918 | 124155 | 4.1 |
| 19 | 28018 | 124837 | 4.5 |
| 20 | 28997 | 124001 | 4.3 |
| 21 | 30543 | 123049 | 4.0 |
| 22 | 28218 | 126658 | 4.5 |
| 23 | 29385 | 124805 | 4.2 |
| 24 | 30584 | 123423 | 4.0 |
| 25 | 27845 | 124450 | 4.5 |
| 26 | 28940 | 124034 | 4.3 |
| 27 | 30208 | 124027 | 4.1 |
| 28 | 28385 | 122841 | 4.3 |
| 29 | 28433 | 123103 | 4.3 |
| 30 | 30533 | 121013 | 4.3 |
| 31 | 27915 | 123111 | 4.0 |
| 32 | 28506 | 121761 | 4.3 |
| 33 | 30009 | 120658 | 4.0 |
| 34 | 27952 | 125076 | 4.5 |
| 35 | 28965 | 124664 | 4.3 |
| 36 | 29576 | 125271 | 4.2 |
| 37 | 28320 | 122473 | 4.3 |
| 38 | 29687 | 121671 | 4.1 |
| 39 | 30656 | 120512 | 3.9 |
| 40 | 27927 | 121437 | 4.3 |
| 41 | 29308 | 118175 | 4.0 |
| 42 | 30701 | 120573 | 3.9 |
| 43 | 27271 | 125549 | 4.6 |
| 44 | 29181 | 123303 | 4.2 |
| 45 | 30332 | 123921 | 4.1 |

**Appendix B. Geographic Spread by Dosing Source**

Simulation of geographic spread of HealtheRx information via clinical dosing and social dosing using the CommunityRx agent based model, by delivery mode. Agents who received clinical dosing could also receive social dosing. The base layer of this map was obtained from Stamen Maps available at [https://stamen.com/open-source/](https://urldefense.com/v3/__https:/stamen.com/open-source/__;!!MvNZe7V6M35iZPhbgng-hfU!hMIS7O2seX9vp_1LOBpg2N9gYEBcAPfYs7rKYAWTmm5M7D_CsQSoXzUAG9Y7k6B4tnm3jP4lVQ$).


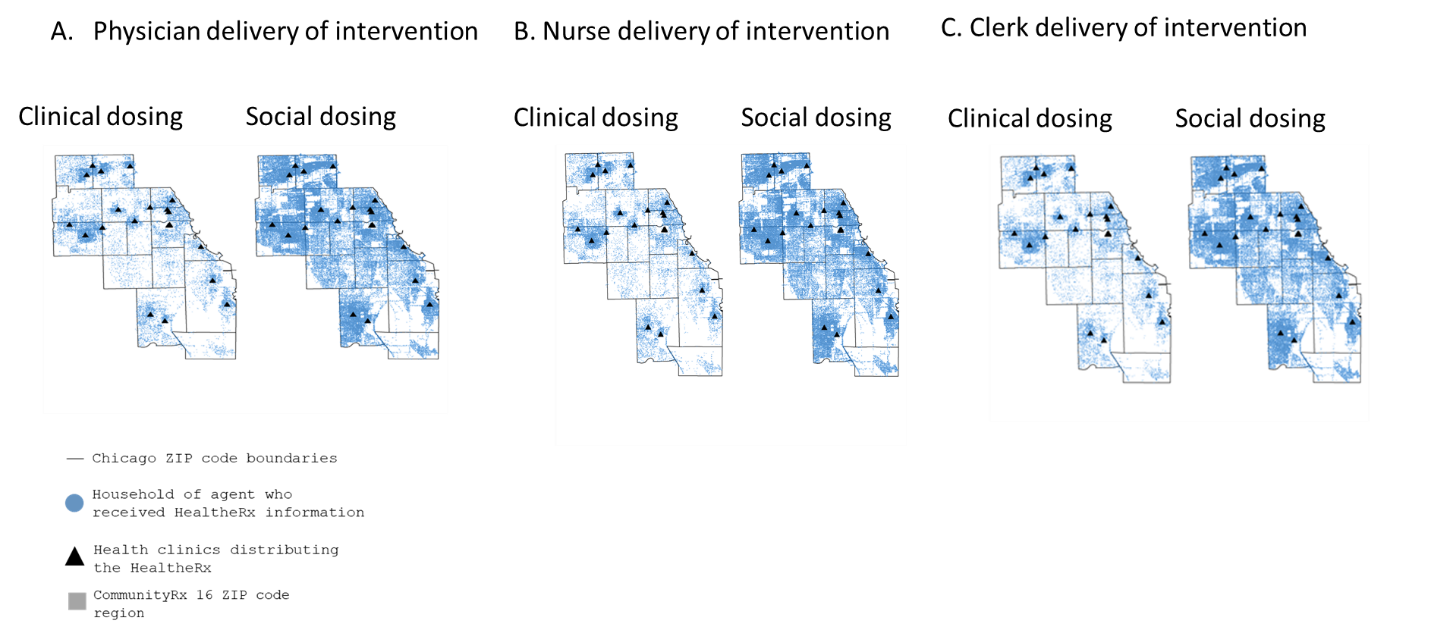

Supplement: S1 Text — Table A. Agent-Based Model Parameters, Description, Values and Sources of Values. Table B. Demographic Characteristics of the Synthetic Population (N = 802,191) Generated Using Data From the Synthetic Populations and Ecosystems of the World; Chicago, IL 2016–2018. Appendix A: Summary Model Description following the Overview, Design concepts and Details (ODD) Protocol (Grimm, 2020). Table C. Common parameter values and ranges for direct versus social dosing experiment runs. Table D. Varied parameter settings experiment runs. Table E. Direct versus Social Dosing Experiment Results. Appendix B. Geographic Spread by Dosing Source. (DOCX) [file pcbi.1009471.s001.docx]
