# Supplementary material for: Building and experimenting with an agent-based model to study the population-level impact of CommunityRx, a clinic-based community resource referral intervention
Source: PLoS Comput Biol. 2021 Oct 25;17(10):e1009471. doi: 10.1371/journal.pcbi.1009471 (PMC8568099; doi:10.1371/journal.pcbi.1009471)
Supplement: Attachment — Submitted filename: PCOMPBIOL-report.pdf [file pcbi.1009471.s002.pdf]

PCOMPBIOL-D-20-02061

This is a highly confusing submission that tells a lot without any clarity as to what the target of this study is. For example, it is not exactly clear to me what this CommunityRX algorithm is supposed to do – is it a disease tracking algorithm? Is it supposed to trace exchanges in the public domain? Is it an information analyser? The abstract does not clarify that and neither the main text.

The github-link leads to the following description:
"In this paper, we describe an agent-based simulation modeling framework applied to Chicago, called chiSIM (for the Chicago Social Interaction Model). Each person residing in Chicago is represented as an agent in chiSIM; all places where people can be located in Chicago also are represented"
But this is from 2018, referring to earlier papers and an existing modelling framework called chiSIM. Is this then a mere repetition of a minor variant of an existing model?

First line of the summary:
"CommunityRX is an intervention delivered in the clinical setting that provides patients with information about community-based resources they can use to manage their health." – I am still lost. What is different (or better) in this algorithm that a simple online search will not achieve? The claim about its usefulness appears stretched. Hence, I will evaluate this manuscript only as an application of a form of theoretical modelling to analyse social science problems.

Line 108: "Scalable" – Indeed, as this appears a minor extension of an existing model called chiSIM.

Line 223: "Generating agent activities" – what 'activities'? What is the algorithm used in this 'model'? Still unclear!

Lines 460-463: The claim is that patients identified new clinical pathways. This sounds preposterous. Were these new pathways discussed with the clinicians and they agreed? Also, are we to believe that the discovery was made by the CommunityRX algorithm independent of intervention by medical experts? This gives the impression of symptomatic google search by laymen that is almost definitely likely to convince an unsuspecting patient that (s)he is suffering from every possible ailment in the Materia Medica!

Overall, I am very uncomfortable with the idea that this CommunityRX is some sort of exploratory pre-panacea that can tell patients what to do, where to look for, and possible therapeutics even. Even if we are to believe that this can be subjectively true for specific disease forms, given the multidimensional and variational nature of chemical pathways for different ailments, a single solution is highly unlikely to work for all. So, the basic premise seems at fault.

Where is the risk management part of this algorithm? Have the results been tested against real medical prescription, separately for multiple ailments?

In their present form, Figures 1-3 are unreadable. Figure 4 contains no description. Is this some sort of clustering identifying behavioural modules against disease forms? Figure 5 looks like an ECG. What is the conclusion drawn from this? Given the rather stochastic nature of the profile, are we to understand that CommunityRX incorporates stochastic modules as well? I am pretty sure that Figure 6 can be data collapsed on to the same plot after suitable rescaling. If this is correct, what conclusion should be drawn from this?

Overall, the list of suspect and omissions is long. But I am willing to give a benefit of doubt to the authors under the assumption that something detailed has been accomplished but not explained clearly. **I suggest a major revision and resubmission, only if the following questions are all answered to satisfaction**:

1. What exactly is the mathematical formulation of the algorithm?
2. Is this is a disease tracker, or a follow-up lead?
3. Does it use any specific form of data modelling, e.g. Machine Learning or Deep Learning?
4. What has this CommunityRX software got that a patient cannot do from a simple browser (e.g. Google) search?
5. How is CommunityRX different from chiSIM and HealtheRX?
6. What is the accuracy level of prediction and how is that validated against complementary results?

The list seems long enough for this submission to be retrievable.
