# Supplementary material for: Building and experimenting with an agent-based model to study the population-level impact of CommunityRx, a clinic-based community resource referral intervention
Source: PLoS Comput Biol. 2021 Oct 25;17(10):e1009471. doi: 10.1371/journal.pcbi.1009471 (PMC8568099; doi:10.1371/journal.pcbi.1009471)
Supplement: Attachment — Submitted filename: Lindau et al Response to Reviewers FINAL.pdf [file pcbi.1009471.s005.pdf]

THE UNIVERSITY OF CHICAGO
DEPARTMENT OF OBSTETRICS AND GYNECOLOGY
THE CHICAGO LYING-IN HOSPITAL

STACY TESSLER LINDAU, M.D., M.A.
Professor of Obstetrics and Gynecology

email: slindau@bsd.uchicago.edu

MC 2050
5841 SOUTH MARYLAND AVENUE
CHICAGO • ILLINOIS 60637
(773) 834-8986
FAX (773) 702-0840

September 10, 2021

Dear Editors Althous and Fefferman,

Thank you for the opportunity to address the remaining critiques of our manuscript "Building and experimenting with an agent-based model to study the population-level impact of CommunityRx, a clinic-based community resource referral intervention" PCOMPBIOL-D-20-02061R1.

As requested, our responses to Reviewer 2 are detailed below, including a description of the significant revisions we have made to the manuscript.

We appreciate the thoughtful review, the constructive process, and the opportunity for further consideration. We do believe this manuscript will be of high value to your readership and the scientific community more generally.

Best,

Stacy Lindau

Authors' Response to Reviewer's Critique:

Reviewer #2: The revised version of the manuscript is much clearer than the previous version and I appreciate the authors' efforts to follow the reviewers' suggestions. However, the manuscript would still benefit from more comprehensive presentation and interpretation/discussion of the in silico experiments' results. And as this paper is intended to serve as an inspiring example, it is crucial to discuss the generalizability of the results of these experiments.

Unfortunately, some of my previous comments/ questions were not sufficiently answered.

1) Methods Sections 3.5.2 and 4.3, Results: Were the outputs of the first two weeks discarded which were influenced by the initialialization of the agents ("burn-in period")? Were only the outputs of following 4 (or 2?) simulated weeks considered for generating the results (e.g. to calculate the average number of minutes per week spent doing each activity? Please clarify.

Authors: Yes, the outputs from the first 2 weeks of the simulation were not considered for calculating any outputs, as this was the burn-in period to obtain model stability. In the manuscript, we use outputs from week 3 of the simulation to report generated results – e.g., Fig 8, and Supplement Appendix 2. Outputs for week 4 show a similar result to week 3. We added the following in section 3.5.2: "Results reported in this paper use output from week 3 of the simulation." We also added the following in section 4.3: "We used week 3 outputs to report results (the first two weeks were considered as the burn-in period for the simulation)." We hope this clarification addresses the reviewer's concern.

2a) Methods Section 3.5.3 Agent information sharing behaviour: Did all agents that were at the same place at the same time share their information? (If 10 persons were at a place at the same time, would any of them receive information about all resources known by all other 9 persons? - Or was some kind of random matching of subsets of present persons performed?

Authors:

Thanks for pointing out that this explanation needed clarification. We edited the following sentences in Section 3.5.3. "Agents at a location share information with other co-located agents based on a threshold defined by the propensity for resource information sharing during that activity and individual random draws against that threshold. The characteristics of agents receiving information were not a factor in the information sharing dynamic. The design of this mechanism was chosen to reflect the fundamental information sharing dynamic commensurate with the expected propensity of resource information sharing determined with expert opinion, and can be considered as a limitation imposed by the data on the model.."

2b) lines 511-512: As far as I understand, only the characteristics of the receiver of information were included (p-score), but not of the donor of information. Then "receiving" should be replaced by "providing" or "giving" in this sentence.

Authors: Only the characteristics of the activity in which the giving agent was engaged when co-located with others was used to determine the propensity for information sharing. We chose to use the word “receiving” to describe this information sharing dynamic, as the agent receiving the information updates their respective β_j^t scores (whereas the giving of information does not change the giving agent’s β_j^t scores).

3) The manuscript still lacks a discussion of the strong restriction that an agent could only conduct an activity that includes "physical and mental health maintenance" if she/he decided to use a rescource (Decision A), otherwise she/he would continue doing the previous activity in their schedule (Decision B) (lines 531-535). As I already mentioned in my review of the previous manuscript version, this restriction of the model might lead to a significant overestimation of the impact of providing information and of information sharing. Therefore, this model restriction, the reasons for its inclusion and its potential influence on the model outcome need to be discussed.

Authors: Thank you for providing the opportunity to clarify and respond to this comment. The CommunityRx intervention is fundamentally a resource referral intervention for local health-related resources, not all types of resources. The CommunityRx ABM seeks to track the changes in the level of knowledge about these health-related resources (obtained through peer dosing, direct dosing, and dosing through use of a resource) and the utilization of these resources (affected by characteristics of those resources, including distance to agent location and the inherent difficulty of overcoming inertia to engage in an activity). While we agree that health-related activities like going for a walk or bible studies are possible without using a CommunityRx resource, the tracking of such activities is not within scope of the study. Further, we clarify that this model is not tracking agent health outcomes due to the CommunityRx intervention. Rather, we model the knowledge about and use of resources that are oftentimes used for physical and mental health maintenance activities. We acknowledge this limitation of the current study in revisions to the Discussion.

We include the following lines in section 3.5.4: “The dynamic described in Equation 1 allows us to isolate and measure the effect of information dosing on knowledge about and use of selected resources for health maintenance or promotion activities that were the focus of the CRx intervention. The model does not include other health promotion and maintenance activities occurring at other places, for example going for a walk outside or an informal support group at a home.” We also added to the limitation section the following: “The CommunityRx ABM only accounts for knowledge about and use of local health-related resources that could be listed on the HealtheRx. Health maintenance activities can occur outside of using these resources (e.g., walking in the neighborhood, an informal support group at someone’s home).”

4) My previous comment "The model was validated only against data that was used to inform the

model. This is not really an external validation and may limit the generalizability of the results. Please discuss." was not considered appropriately in the current version of your manuscript. Although Methods Section 3.7 clarifies the calibration and validation process, in the discussion, e.g. lines 747-748, 750-768 (and the Abstract, l. 69-71) any statement on the limited generalizability of the results of the experiment is missing. Instead, the simulation results are presented in a way as if they were generally valid.
Please make your own opinion ("While we calibrate and validate our model against empirical data, we do not claim model generalization beyond recreating the CommunityRx simulation in silico. Our general process of model building and the use of computational laboratories, however, is generalizable.") absolutely clear in the Discussion section and the Abstract.

Authors: We thank the reviewer for highlighting this issue and completely agree that this distinction is important in framing our work. As suggested, we have added the following:

a) In the Discussion section - "Thus, while the focus of the CRx ABM is on simulating the CRx intervention, the process of model building, and computational experimentation presented is generalizable to other large-scale ABMs, for example those modeling information diffusion processes. However, generalizability may be limited because the model was validated only against the data that were used to inform the model building"

b) In the abstract "While the focus of the CRx ABM is to recreate the CRx intervention in silico, the general process of model building, and computational experimentation presented is generalizable to other large-scale ABMs of information diffusion."

Reviewer: Additionally, I found some minor issues:
line 300: "HealtheRxs"

Authors: This typo has been corrected.

line 318: "exchanging"

Authors: This typo has been corrected.

Supplement Table 1: Full citation of "Garibay, 2011" is missing (or is it Garibay, 2014?)

Authors: It is Garibay 2014 and has been corrected. We appreciate your attention to detail.

Reviewer #4: Review is uploaded

Have the authors made all data and (if applicable) computational code underlying the findings in their manuscript fully available?

The PLOS Data policy requires authors to make all data and code underlying the findings described in their manuscript fully available without restriction, with rare exception (please refer to the Data Availability Statement in the manuscript PDF file). The data and code should be provided as part of the manuscript or its supporting information, or deposited to a public repository. For example, in addition to summary statistics, the data points behind means, medians and variance measures should be available. If there are restrictions on publicly sharing data or code —e.g. participant privacy or use of data from a third party—those must be specified.

Reviewer #2: Yes

Reviewer #4: Yes

PLOS authors have the option to publish the peer review history of their article (what does this mean?). If published, this will include your full peer review and any attached files.

If you choose "no", your identity will remain anonymous but your review may still be made public.

Do you want your identity to be public for this peer review? For information about this choice, including consent withdrawal, please see our Privacy Policy.

Reviewer #2: **Yes:** Veit Zoche-Golob

Reviewer #4: **Yes:** Emmanuel de-Graft Johnson Owusu-Ansah

Figure Files:

While revising your submission, please upload your figure files to the Preflight Analysis and Conversion Engine (PACE) digital diagnostic tool, https://pacev2.apexcovantage.com. PACE helps ensure that figures meet PLOS requirements. To use PACE, you must first register as a user. Then, login and navigate to the UPLOAD tab, where you will find detailed instructions on how to use the tool. If you encounter any issues or have any questions when using PACE, please email us at figures@plos.org.

Data Requirements:

Please note that, as a condition of publication, PLOS' data policy requires that you make available all data used to draw the conclusions outlined in your manuscript. Data must be deposited in an appropriate repository, included within the body of the manuscript, or uploaded as supporting information. This includes all numerical values that were used to generate graphs, histograms etc.. For an example in PLOS Biology see here: http://www.plosbiology.org/article/info%3Adoi%2F10.1371%2Fjournal.pbio.1001908#s5.

Reproducibility:

To enhance the reproducibility of your results, we recommend that you deposit your laboratory protocols in protocols.io, where a protocol can be assigned its own identifier (DOI) such that it can be cited independently in the future. Additionally, PLOS ONE offers an option to publish peer-reviewed clinical study protocols. Read more information on sharing protocols at https://plos.org/protocols?utm_medium=editorial-email&utm_source=authorletters&utm_campaign=protocols
