# Supplementary material for: Building and experimenting with an agent-based model to study the population-level impact of CommunityRx, a clinic-based community resource referral intervention
Source: PLoS Comput Biol. 2021 Oct 25;17(10):e1009471. doi: 10.1371/journal.pcbi.1009471 (PMC8568099; doi:10.1371/journal.pcbi.1009471)
Supplement: Attachment — Submitted filename: Lindau et al Response to Reviewers FINAL.docx [file pcbi.1009471.s003.docx]

Dear Editors Althouse and Fefferman,

Thank you for the opportunity to revise our manuscript " Building and experimenting with an agent-based model to study the population-level impact of CommunityRx, a clinic-based community resource referral intervention" for consideration at *PLOS Computational Biology*.

This resubmission is a significantly revised version that takes into account the reviewers' comments. Below, you will find a detailed list of our responses to the review comments and a description of the changes we have made in the manuscript

Sincerely,

Stacy Lindau

**Authors’ Response to Reviewers’ Questions**

**Reviewer #1:**

This article describes the CommunityRX agent-based model (henceforth referred to as the CRX-model) aimed at doing in silico experimentation on an in vivo intervention called CommunityRX. In this intervention information of local health resources is shared with individuals during their touch point with the health system. As individuals had indicated they share such information, the CRX-model is aimed at capturing evidence of the propagating systemic impact of such an intervention (beyond the cohort directly exposed). The manuscript is well written and the method of using ABM to explore both the size and mechanism of the systemic impact has both merit, can provide invaluable for decision support, translates far beyond the setting in which it is current applied, and as such would be of interest to the journal audience.
The self-reported goal of the manuscript is “to describe the interdisciplinary processes and data sources used to develop an ABM to study an information-based health intervention“ (L164/165), yet I believe it falls short of this aim. The model description provided is too high level to fully grasp model functioning or its details and consequently for many of the details the two previous studies on the model are cited [30/31]. If model description is indeed the goal, there are standards such as ODD (Grimm et al. 2006,2010,2020) that would greatly improve the manuscript structure and would provide guidance as to how to accurately do so. The more fundamental question is if model description should in fact be the desired purpose of the manuscript. I would deem a manuscript that only provide a model description as outside the scope of PLOS Computational Biology, and potentially suited for the realm of the contextual health domain. **What would be if interest to the journal audience would be a detailed description of how to build this type of ABM (focused on the method of model building), or an illustration of how such an ABM could provide insight trough in silico experimentation**. While aligning with either of these aims would require mayor revisions there is merit in doing so.

Authors: We appreciate this very constructive critique. We have refocused the paper to describe the model building process and illustrate how an ABM can provide insight about information diffusion through *in silico* experimentation. We have also applied the summary ODD protocol from Grimm et al. (2020), referencing the detailed design papers already published (Kaligotla 2018; Kaligotla, 2020). Also, Supplement Appendix 1 summarizes our model and design features important to interpreting the study results.

Regardless of the revision path chosen, a more detailed model description of the model will be required (either as part of the Supplementary information, or in the main text), and the following comments would support these efforts.
1. Provide an overview of model behaviors, prior to specifying the details (moving section implementing the ABM to the start of the methods section would be a good start).

Authors: We have reordered and expanded the Methods section. Section 1.0 lays out the rationale for building the ABM. Section 2.0 describes and illustrates the interdisciplinary model-buliding process, addressing another reviewer comment. The first paragraph of Section 3.0 provides an overview of the model, including the implementation language that was previously much later in the Methods section. The remainder of Section 3.0 describes the model building and key design features following the summary ODD protocol from Grimm et al. (2020).

2. Include a pseudo code description of the model behaviors to that overview section

Authors: We now describe model processes that occur at every time step in paragraph 3 of Supplement Appendix 1. Additionally, the revised Methods Section 3.4 provides essentially the same detail as a pseudocode would. Further details of model processes are also described in the revised Methods, including Section 3.7 “Model implementation and calibration.” We chose this description over detailed model pseudocode to avoid an overtly technical model description that repeats other published work (Kaligotla 2018; Kaligotla, 2020). We hope this makes for a better flow; thank you for the suggestion.

3. Provide the characteristics of the population (individual characteristics and their distribution)
(note that the above should follow naturally form adopting ODD as the standard for model description)

Authors: In the revised Methods Section 3.3 “CommunityRx ABM Entities,” we describe the synthetic population generally and provide the state variables characterizing the population in Supplement Table 2. In the Results section of the original submission (para 1), and again in this draft, we describe the characteristics of the synthetic population and detail these (age, gender, race, ethnicity) in Supplemental Table 2.

4. The schedule assignment is random (L230 – 234), which has a large randomizing effect on the networks that will be created. While it is debatable if this is realistic, the implications of this modeling decision should be explored, or be made explicit at the least.

Authors: In a revised Methods Section 3.5.1, we address this point. Schedule assignments were based on matching two data sets - the American Time Use Survey (ATUS) and the Synthetic Population dataset (SPEW). The matching was done on the following sociodemographic characteristics: age, gender, race, and ethnicity. We needed to ensure some randomness of activity schedules available to each agent (or else agents follow the same schedules every simulated day). While a larger sample of available schedules does indeed introduce stochasticity, it was deemed better than the case of having groups of agents with a singular or very few schedules, which could result in unrealistically repetitive activity patterns. Future work will focus on the implication of this randomization on the resulting networks.

5. The mapping of ATUS activities and CRX resources should be clarified, currently it is not clear from the text how exactly this works, and what the scope of activities are. More details are needed.

Authors: We have included additional detail about the ATUS activity and HealtheRx service type mapping procedures in the revised Methods Section 3.5.1, paragraph 3.

6. Clarify the information sharing process in more detail (see comment below for details. An general modeling concern relates to the specification of the information sharing mechanism in the CRX-model. The authors signal the awareness of the notion that information transfer to consist of three steps (sending information, transmission, and reception) by stating the steps required in the vivo intervention result in administration of the intervention (L122-131). In each propagating process there will be the action of sending, the medium transmitting that signal, and a reception processes by which the recipient processes the signal (and updates their state or behavior). While these are touched upon for the initial transfer during the in vivo intervention, for consequent information sharing among peers this is not the case. In fact you based the notion of secondary information transfer solely on the account that a respondent have indicated they shared information, without evidence that behavior is changed as a result of this sharing the presumed systemic effect is in fact speculative. As far as I can tell form the manuscript the steps following sharing are (transfer and reception) are assumed to occur as a given. What is more is seems a if sharing occurs in a broadcasting manner, touching all who are collocated. As the secondary spread is the sole driver of the presumed systemic impact a more detailed elaboration of such assumptions is required for this model.

Authors: We have substantially edited the organization of the Methods section to provide more clarity. We describe the information sharing behavior process (3.5.3) before describing the agent decision-making process (3.5.4). We also clarify the assumptions in the relevant subsection.

Sharing and of information in the CRx ABM depends on the activity being performed at a given location. For some activities (like sleeping, for example), no information is shared, while for others, varying levels of information sharing can occur. When agents are co-located, based on the propensity of information sharing associated with an activity, agents will exchange information. As a result of the information being shared, all co-located agents’ beta scores are updated (via a social dosing effect). The change in beta scores affects their future decisions on healthful activities that require the AB decision as modeled. These features of the model are described in more detail in revised Methods Section 3.5.3 and 3.5.4.

The modeling choice of ‘broadcast sharing’ among co-located agents subject to activity being performed is indeed an assumption. We address this limitation at the end of Methods Section 3.5.3. We believe this information sharing dynamic captures the peer dosing effect vis-à-vis direct dosing effect. The parameter values for these effects were critical to model calibration and described in detail in Kaligotla, 2020.

Going back to the revision paths; If the goal will be to present the process of model building in detail there are concerns relating to the overlap with earlier manuscripts, specifically [31]. It will need to be made evident what is novel in the current submission.

Authors: Per above, the paper is now focused on (1) describing the model building process, which is distinct from the previously published model description Kaligotla 2018 and model calibration and validation Kaligotla, 2020; and (2) illustrating how an ABM provides insight about information diffusion through in silico experimentation.

Alternatively, If the goal will be to present the use of this model for decision support or even learning about the mechanisms of secondary spread the outcomes will need to be refined. In fact apart from the degree distribution the results presented in the current manuscript are in fact input data rather than model outcomes. What is more, the degree distribution of the spread network might not be the most informative outcome. Comparisons of community resource use under different rates of spread, network size (both in terms of edges and vertices) might allow you to drive home points relating to reach and impact on behavior much better.

Authors: We have added to the revised Methods a section on *in silico* experimentation (Section 4.0) and describe the results of one delivery mode experiment as an example of how the model can be used to complement clinical trial methods. The reviewer’s suggestions about other relevant experiments anticipate our future work.

Lastly, as a minor comment I would add that the clarity of figures would benefit from higher resolution images.

Authors: Per our communication with the editorical team, the poor resoluation was a result of the reviewer receiving a compressed PDF. This should not be an issue with the final publication. Please let us know if we need to provide files in a different format.

**Reviewer #2:** The authors have submitted a very interesting manuscript about an advanced topic. The integration of (clinical) trials and systems science methods for studying population health impacts of (medical) interventions is a combination that will become more common in near future. In their manuscript they clearly describe an example of the process of this integration which can guide others.

However, I found some issues where the manuscript needs improvement before it meets the expectations of a good model of this integration process.

First, a manuscript describing a complex agent-based model should follow generally accepted reporting guidelines like the ODD Protocol for Describing Agent-Based and Other Simulation Models (DOI: 10.18564/jasss.4259).

Authors: This comment, also raised by R#1, has been addressed.

Comments in detail:

line 135-136: "tens of thousands"

Authors: This typo has been corrected.

line 221: Where were the characteristics described? Was the synthetic population static or dynamic?

Authors: The sociodemographic characteristics of the synthetic population were described in Supplemental Table 2 and the first paragraph of the Results section. These characteristics were static. This point has been added to the description of how we built the CommunityRx ABM Entities in the revised Methods Section 3.3 CommunityRx ABM Entities (paragraph 2).

line 231: The schedules were randomly assigned. Did you include differences between weekdays, weekly and seasonal influences on the daily schedules? If not, please discuss the reasons.

Authors: Weekdays and weekends were indeed distinct in our model. This point has been clarified in the manuscript (revised Methods Section 3.5.1 Agent Activity Behavior). We, however, do not account for weekly and seasonal schedules due to the limited availability of such data. This is a limitation of our model that has been described in the manuscript (also Methods Section 3.5.1 Agent Activity Behavior para 2).

Generating agents' decisions:
- You included a very strong restriction in the model by forbidding agents to conduct activities matching CommunityRx resources if they did not use such a resource (i.e. not doing the activity without using a resource). Activities like going for a walk or bible studies might be possible without using a CommunityRx resource in reality. I think this strong restriction of the model might lead to a significant overestimation of the impact of providing information and of information sharing. Please discuss.

Authors: Agents can indeed conduct activities that do not use a CommunityRx resource. To clarify this point, we detail in Methods Section 3.5.1 Agent Activity Behavior (para 3) two subsets of activities. One of these subsets include only physical and mental health maintenance activities that involve CommunityRx resources. Only these specific activities are subject to the AB decision process. For activities that are not classified as health maintenance activities, the AB decision model is not applied.

- Information about a resource was modeled to have an enhancing effect only - not a negative one, which can be also possible in reality. Please discuss.

Authors: The only negative effect we account for is in the decay parameter – where the agents can ‘forget’ about a specific resource. We did not include a negative social dosing effect (e.g. negative information such as “don’t go to that place,” or misinformation). Interestingly, empirical studies have not elicited any reports of sharing negative or misinformation. Of course, we agree with the reviewer, that this is possible and quite interesting. We have revised the manuscript to address this issue in the Introduction (para 2), and Methods Sections 1.0 Rationale for building the CommunityRx ABM, and 3.5.2 Agent knowledge evolution.

Generating agents' information sharing behaviors and knowledge evolution:
- Did you consider a kind of "burn-in" period? How long did it take until no effects of the arbitrary initial values were observable any more?

Authors: Yes. The initialization process was described in the Methods Sections “Generating agents’ information sharing behaviors” and “knowledge evolution.” In the current draft, initialization is discussed in Methods Section 3.5.2, Agent knowledge evolution. The first two weeks were considered as a burn-in period for the entire simulation. We generally observed stabilized behavior by week 3 of the simulation.

- The necessary interaction between the donor and receiver of information about resources is not sufficiently described, e.g. how was determined, which information (information about which resources) was shared? Only the propensity of the receiver is mentioned, but no properties of the donor.

Authors: We have added the following to Methods Section 3.5.3 Agent information sharing behavior to clarify: “Co-located agents shared information on all of the 200 resources each agent maintained. The characteristics of agents receiving information were not a factor in the information sharing dynamic. This modeling choice was made to avoid an overly complicated sharing dynamic and is a limitation of our model.”

- line 351: Where were the validation results described?

Authors: Model calibration and validation were described in detail in previously published work (Kaligotla, 2020) and hence not included in detail here (an overview is provided in 3.7 “Model calibration and validation”). We clarify this in the manuscript.

Results:
- Which simulated time period was used to get the results?

Authors: Methods Section 3.4 “CommunityRx ABM spatial and temporal resolution” now states: “Each time step in the model represents one hour of simulated time and simulations are run for a period of at least 4 weeks. We generally observed stable behavior by week 3 of the simulation.” In Methods Section 4.3 “Experiment to determine optimal mode of CRx intervention delivery,” we describe “4 week long simulations, with 15 runs for each of 3 HealtheRx delivery modes.”

- The model was validated only against data that was used to inform the model. This is not really an external validation and may limit the generalizability of the results. Please discuss.

Authors: We discuss the calibration and validation process in previous work (Kaligotla, 2020). Methods Section 3.7 “Model calibration and validation” describes how we calibrate and validate the model: “we selected resource use in our agent population for 10 specific resources (clinics) that required an A/B decision and calibrated our parameter set where the model output (average resource use) was within the range of empirically observed clinic visits. A random forest model was iteratively fitted to model evaluations to characterize the model parameter space against observed empirical data.” While we calibrate and validate our model against empirical data, we do not claim model generalization beyond recreating the CommunityRx simulation *in silico*. Our general process of model building and the use of computational laboratories, however, is generalizable.

Discussion:
- lines 483-485: These were design descisions, i.e. this behavior of the model was expected. Something would have been wrong in the implementation if the model did not show this behavior. Please clarify.

Authors: Yes. This was indeed an internal test for model behavior. Agents’ resource knowledge did indeed evolve as a function of our model design. This sentence was included to show that the model did perform as designed and expected. Additionally, we calibrate and validate the model on these parameter values, thus, showing model behavior was important. This also helps us demonstrate the effects of social dosing and dosing decay on knowledge evolution and resource use behavior at the population level.

**Reviewer #3**

This is a highly confusing submission that tells a lot without any clarity as to what the target of this study is. For example, it is not exactly clear to me what this CommunityRX algorithm is supposed to do – is it a disease tracking algorithm? Is it supposed to trace exchanges in the public domain? Is it an information analyser? The abstract does not clarify that and neither the main text.

Authors: The CommunityRx algorithm uses data from patients’ medical records, including age, gender, race/ethnicity, home address, preferred language, and medical diagnoses, to match patients to a list resources near their home. The algorithm does not trace exchanges in the public domain. We have added text to the Introduction (paras 3, 4) and a figure (Figure 2) to clarify what is meant by the CommunityRx algorithm. We also reference the algorithm in revised Methods Section 3.6 CommunityRx ABM submodel: delivering the HealtheRx in silico.

The github-link leads to the following description: “In this paper, we describe an agent-based simulation modeling framework applied to Chicago, called chiSIM (for the Chicago Social Interaction Model). Each person residing in Chicago is represented as an agent in chiSIM; all places where people can be located in Chicago also are represented” But this is from 2018, referring to earlier papers and an existing modeling framework called chiSIM. Is this then a mere repetition of a minor variant of an existing model?

Authors: This revised manuscript is describing how we built and conducted *in silico* experiments with an ABM (CommunityRx ABM), which was developed using the chiSIM framework. chiSIM is a framework that provides the capabilities for building large-scale ABMs (see the figure below, Figure 7 in Macal 2018). We explain in the manuscript (both Abstract and Section 3.0 of the revised Methods) that the CommunityRx ABM was implemented in C++ using Repast for High-Performance Computing ABM toolkit and the Chicago Social Interaction Model (chiSIM) framework (Collier 2013; Macal, 2018; Collier 2018; Tartara, 2017). As detailed in the revised Methods section, the CommunityRx ABM includes definitions of dynamic agent activity behaviors, agent decision making, and other features that was purposefully built to model the CommunityRx intervention *in silico*. All these represent a development from chiSIM.

First line of the summary: “CommunityRX is an intervention delivered in the clinical setting that provides patients with information about community-based resources they can use to manage their health.” – I am still lost. What is different (or better) in this algorithm that a simple online search will not achieve?

Authors: The text describing the algorithm has been revised for clarity, as noted above. In addition, we have added substantially to the Introduction and Methods sections to explain the CommunityRx intervention and the evidence related to it. CommunityRx was created, in part, due to systematic deficiencies in Google and other mainstream data sources which under-represent open and operating businesses and organizations in higher poverty communities (Makelarski, 2013). These deficiencies impede the efforts of healthcare professionals to meaningfully execute on clinical best practice guidelines that indicate a wide range of community resources for various health conditions. In contrast to a simple online search for “food support,” for example, the CommunityRx algorithm draws on individual data in the electronic medical record (e.g. patient age, home address, preferred language, medical conditions) to return a set of community-based resources that match these characteristics. A Spanish-speaking person with diabetes would be sent to fitness, diabetes management, eye care and foot care resources offered in Spanish, for example.

The claim about its usefulness appears stretched.

Authors: We have added text in the Introduction to elaborate on evidence about the efficacy/effectiveness of the intervention, which includes data from a study with >113K participants and a clinical trial of 411 middle-age and older adults (Lindau, 2016; Lindau, 2019). We have also added another relevant paper that has been provisionally accepted for publication, pending minor final edits (Lindau, 2021).

Hence, I will evaluate this manuscript only as an application of a form of theoretical modelling to analyse social science problems. Line 108: “Scalable” – Indeed, as this appears a minor extension of an existing model called chiSIM.

Authors: The term “scalable” is used in the Introduction section to describe the CommunityRx intervention. The intervention was proven scalable when it was rapidly deployed into 33 unique healthcare sites (integrating with 3 different electronic medical record systems) and a wide range of workflows over ~24 months (as shown in reference Lindau, 2016). Today, as the result of technology transfer to a private entity, the CommunityRx system is operating in 15 U.S. states with more than 90 unique partners serving several million patients.

To address the comment regarding “a minor extension of an existing model called chiSIM”: As described in the text – and clarified thanks to comments especially from R1 -this manuscript presents CommunityRx as a case study for applying computational methods like agent-based modeling to complement knowledge that can be generated using traditional clinical trial design. Our model recreates the CommunityRx informational intervention so its impact can be studied at the level of a large, predominantly African American urban region with >800K residents (802,191 agents). As previously discussed (in our response to R3’s second comment), the CRx ABM includes several extensions to the chiSIM framework.

Line 223: “Generating agent activities” – what ‘activities’?

Authors: The revised manuscript uses Grimm et. al. summary ODD method (2020) to better organize the methods. Section 3.5.1 “Agent activity behavior” describes the process by which we assign activity schedules to agents in our synthetic population.

What is the algorithm used in this ‘model’? Still unclear!

Authors: This question is addressed above.

Lines 460-463: The claim is that patients identified new clinical pathways. This sounds preposterous. Were these new pathways discussed with the clinicians and they agreed?

Authors: We believe the reviewer is referring to this statement from the Discussion:

In prior studies,[6,7] we discovered that nearly half of patients and clinicians who received the intervention shared information about resources with others, identifying a potentially potent but previously unexplored pathway through which a clinic-based intervention can impact population health.

We have revised the statement for clarity:

In prior studies,[6,7] we discovered that nearly half of patients and clinicians who received the intervention shared information about resources with others, providing evidence of a potentially potent but previously unexplored pathway through which a clinic-based intervention can impact population health.

We did not intend to suggest that patients themselves identified new clinical pathways. Rather, empirical studies of patient outcomes related to the intervention point to a pathway through which the intervention may yield impact.

Also, are we to believe that the discovery was made by the CommunityRX algorithm independent of intervention by medical experts? This gives the impression of symptomatic google search by laymen that is almost definitely likely to convince an unsuspecting patient that (s)he is suffering from every possible ailment in the Materia Medica! Overall, I am very uncomfortable with the idea that this CommunityRX is some sort of exploratory pre-panacea that can tell patients what to do, where to look for, and possible therapeutics even. Even if we are to believe that this can be subjectively true for specific disease forms, given the multidimensional and variational nature of chemical pathways for different ailments, a single solution is highly unlikely to work for all. So, the basic premise seems at fault.

Authors: Reviewer questions regarding the CommunityRx algorithm are addressed above and in the revised manuscript. We believe the clarification renders the concerns about a panacea and the variational nature of chemical pathways irrelevant. Of note, several co-authors, including the first author, are practicing physician scientists who have developed and tested the CommunityRx intervention over more than a decade. As explained above and in the manuscript, CommunityRx prescribes community-based resources to support wellness and disease self-care. CommunityRx does not prescribe medications or medical treatments.

Where is the risk management part of this algorithm? Have the results been tested against real medical prescription, separately for multiple ailments?

Authors: We have clarified that the CommunityRx algorithms are generated based on extant literature with expert and patient or community advisor review. As explained above, and in further revisions to the manuscript, these algorithms consume extant demographic and health condition data in an individual patients’ electronic medical record in order to match those data to relevant community-based resources located near the patient’s home address. This approach overcomes the enduring and endemic problem in healthcare that results from doctors and other clinicians regularly recommending patients make use of community resources (e.g. to lose weight, get fit, stop smoking) but with no reliable information about where patients can go to do these activities and for which activities they are eligible. In the U.S., most of these health promoting activities, especially for lower income people, are supported by non-profit, non-government organizations. Each organization has a different set of eligibility criteria. Patients’ efforts at self-care and disease self-management are often stymied by lack of poor quality information about these resources. CommunityRx is designed – together with the communities we serve – to overcome these barriers to good health. Hundreds of thousands of patients across multiple geographies have received one or more HealtheRx. To our knowledge, there have been zero instances where risk management concerns were raised.

In their present form, Figures 1-3 are unreadable. Figure 4 contains no description. Is this some sort of clustering identifying behavioural modules against disease forms? Figure 5 looks like an ECG. What is the conclusion drawn from this?

Authors: Per our communication with the editorial team, the poor resolution was due to the reviewers receiving a compressed PDF. This problem should not be an issue in the final publication, but please let us know if you need any alternative versions of our submitted figures. The Figure 4 referenced by the reviewer is now Figure 6 in the current version. This figure depicts the visualization of network pathways through which agents, stratified by age (16-25 years = orange, 65+ years = purple) exchange information about resources. The legend for this figure is in the results section, after paragraph 4. The description of how Figure 5 was generated is in revised Methods Section 3.5.2 “Agent Knowledge Evolution.” Figure 5 presents an exemplar instance of the evolution of three agents’ knowledge (Beta, β) of eight resources over time.

Given the rather stochastic nature of the profile, are we to understand that CommunityRX incorporates stochastic modules as well?

Authors: Although we are not entirely clear what is being asked here, the *in vivo* CommunityRx intervention does not incorporate stochastic modules. The HealtheRx is generated based on characteristics of individual patients using the evidence-based algorithms previously described. Issues of stochasiticity that limit the ABM are addressed above an in the revised manuscript (e.g. Methods Section 3.5.1 Agent activitybehavior, para 2).

I am pretty sure that Figure 6 can be data collapsed on to the same plot after suitable rescaling. If this is correct, what conclusion should be drawn from this?

Authors: The Figure 6 referenced by the reviewer is now Figure 7 in the current version. Figure 7 shows the differences in degree distributions of the endogenous co-location networks (information pathways) that result from our ABM as a function of different rates of dyadic exchange. Essentially, we demonstrate here that the networks via which information flows and spreads in a population are endogenous to the model.

Overall, the list of suspect and omissions is long. But I am willing to give a benefit of doubt to the authors under the assumption that something detailed has been accomplished but not explained clearly. I suggest a major revision and resubmission, only if the following questions are all answered to satisfaction:

1. What exactly is the mathematical formulation of the algorithm?

Authors: We interpret this comment to mean the mathematical formulation of the Community Rx ABM. The ABM consists of mathematical as well as rule-based dynamics that have been clarified in the manuscript. We additionally also include a supplement (Supplement Appendix 1) with a summary ODD model description. We note that vocabulary used in this interdisciplinary work, specifically reference early in the paper to a software algorithm used by CommunityRx to match people to community resources, seems to have caused major confusion for this reviewer. Because: (1) the other reviewers did not share this concern, (2) we have further illustrated and explained what is meant by the CommunityRx algorithm and (3) this term is used in other published literature pertaining to the CommunityRx intervention, we did not remove the term.

2. Is this is a disease tracker, or a follow-up lead?

Authors: We are not clear on this question. If the question is whether CommunityRx is a disease tracker or a follow-up lead, it is not a disease tracker and we are unclear regarding what is meant by “follow-up lead.” It is a community resource referral tool that uses evidence-based algorithms to match people to resources suited to their demographic characteristics and health conditions. We have provided more detail on the CommunityRx intervention and algorithm above and in the manuscript.

3. Does it use any specific form of data modelling, e.g. Machine Learning or Deep Learning?

Authors: The CommunityRx intervention does not use machine learning or deep learning. A more detailed description of the intervention and the related matching algorithm (it matches people, based on their characteristics, to resources in the community for which they are eligible) has been provided above and in the manuscript.

4. What has this CommunityRX software got that a patient cannot do from a simple browser (e.g. Google) search?

Authors: This question has been addressed above and elaborated in the manuscript.

5. How is CommunityRX different from chiSIM and HealtheRX?

Authors: CommunityRx is the name of the intervention that is used in healthcare practice to enable automated and personalized referrals for patients to community resources that are relevant to their health conditions, near their home and for which they are eligible. This information has been clarified above and in the manuscript. The CommunityRx system generates a “HealtheRx,” or printed resource referral list for each patient. An example of a HealtheRx is included in the Introduction (Figure 1). The HealtheRx is printed in the clinic and can also be emailed or texted to the patient. The HealtheRx is a document that includes the personalized list of community resources generated for the patient at their visit. chiSIM is an agent-based modeling framework that enables large-scale computational experimentation at scale.

6. What is the accuracy level of prediction and how is that validated against complementary results? The list seems long enough for this submission to be retrievable

Authors: The CRx ABM was not designed per se to predict behavior. That said, we have addressed model calibration and validation in the manuscript (revised Methods Section 3.7 “Model imnplementation and calibration”). We also make clear that a comprehensive description of the model calibration and validation process has been previously published Kaligotla, 2020.

**Reviewer #4:** The following are my general comments of the article
The article shows a coherent story of the study, the authors have taken the time to explicitly explained the whole concept of Agent Based Modeling and its associated procedures. The arrangements were made in such a way that, it predominantly centered on language understandable by all thus non-technical interpretations were offered.

In as much as the reviewer believes detailed procedures and well explained methodology and implementation understandable by ordinary people with interest in science, the author also suggests the following below for the authors to reconsider in providing further explanations to make the concepts much clearer.

Report on Reviewed article
COMMENT: Line 133 The authors should consider deleting line 133 sentence as it doesn’t add up to the sentence. It can be placed at where funding information is required to be place
133 With initial funding from a Center for Medicare and Medicaid Innovation award (3/2013-5/2015,
134 “CRx-1”),

Authors: We have made this edit.

COMMENT: Line 135 I believe the authors were referring to tens of thousands instead of "thens" of thousands 135 30 month period. All patients living in the target geography were eligible, including thens of 136 thousands of people with cancer, cardiovascular disease, pulmonary disease, obesity and other

Authors: The reviewer is correct. We have made this edit.

COMMENT: Line 137 Authors should include the unit of measurement of age, “18-93 years”
137 common chronic conditions. In a telephone survey of 458 HealtheRx recipients ages 18-93,

Authors: We have made this edit.

COMMENT: Line 197 This statement should be appropriately recapture and the link should be inserted as part of the appendix. Moreover, authors should clearly show which part is in a bracket and which is not, the end of the link has a close bracket but the beginning of the bracket is not seen.
197 toolkits. The model code and the workflow code used to implement the parameter space
198 characterization experiments are publicly available at [https://github.com/jozik/community-rx](https://urldefense.com/v3/__https:/github.com/jozik/community-rx__;!!OaZRxjSJGsACmqMyc8s!eVoXBGJM7ZXCg3gU50BWVqwJoKqbwpghUpZTPJIGLWX-0syJLJ5phyIntfIx6CmSZSJirg$)).

Authors: We have fixed the bracketing in the manuscript.

COMMENT: Line 250. What informed this categorization, any special reasons? as why not other age groups different from what you have chosen?
250 was computed within each of four age groups (<31, 31-45, 46-65 and 65+ years), weighted by

Authors: We have addressed the age group question with the following statement in the revised Methods Section 3.5.1 “Agent activity behavior”: “These groups were defined using demographic, life course and healthcare policy considerations and are consistent with commonly applied strata for age in the clinical and population health literature.”

COMMENT: Line 276: Can authors throw more light as to how (inherent inertia) this was captured or measured? As it could be a source of uncertainty in the model itself? Was it also derived from Skolasky et al?
276 iii) the inherent inertia that they needed to overcome for using that service (γj).

Authors: Agent inertia was parameterized into Low, Medium, and High based on the difficulty associated with performing an activity at a resource. The assignment of low, medium, or high was done through expert informant surveys completed by a diversity of researchers with expertise about the geographic region and its population. The parameter values for gamma (inertia) values were set during model calibration runs, ensuring that model output matched empirical visits. Details on the calibration process are available in previously published work (Kaligotla, 2020).

COMMENT: Line 305/308: In equation presentation, variables/parameters, constants are explained just below the equations, in equation 2, since all variables have been previously explained, could authors given meaning to the value, εx, where ∈?. Moreover, εx could have been any value, why the chosen values on line 308?
305: of resource information was associated with a value, εx, where ∈
308: used to produce Figure 5, the following values were used = {0.05, 0.15, 0.25, 0.2, 0.9, 1}.

Authors: The relative values for Figure 5 were informed by expert informants and through sensitivity analyses. The goal was to reproduce the evolutionary dynamics of resource information knowledge at an agent level. Epsilon values for the different dosing sources (x) are set between 0 and 1 to ensure that the Beta score remains between 0 and 1. Indeed, a range of values for each epsilon(x) is possible, with each parameter value dictating the relative weights for sources of information. As a modeling choice, we set the relative epsilon values for the different sources, i.e., dosing from a doctor is weighted higher (lower epsilon score) than dosing from a peer (higher epsilon score). Values for some of these parameters (dosing decay and peer dosing) were set during the calibration process described in Kaligotla, 2020

NOTE (Not necessarily a required consideration, but a simple explanation could help, on below general comments)
1. It is widespread believed that, the alternative version of ABM could be made in differential equations since its well suited in dynamical systems of how agents behave (though the debate has not settled yet within the mathematical science fraternity), could the authors consider explaining why ABM is well suited for this study but not any of the Complex Adaptive Systems Modeling techniques (Complex Network Modeling Level, Exploratory Agent-based Modeling Level, Validated agent-based modeling and Descriptive Agent-based Modeling )?

Authors: An ABM approach allows us to model behaviors at an individual agent level while considering heterogeneous agents, something that becomes exceptionally challenging while using the differential equations approach. Furthermore, an ABM approach allows us to model heterogeneous agent behavior to result in endogenous colocation networks (that indicate information pathways). This approach enables us to tie in individual choice behavior to emergent population-level outcomes (like total visits to a clinic or other health maintenance activities performed). The micro-to-macro level dynamics make ABMs particularly well suited to study an informational intervention.

2. The GitHub link provided by the author: Authors should consider adding in-depth comments to the scripts provided in the GitHub in order for users who might "clone the scripts" be able to understand what the lines of codes were meant for, for the sake of reproducibility of work which is the cornerstone of today's scientific study dissemination.

Authors: We agree with the reviewer and have provided additional references and guidance in the Github repository.

**Have all data underlying the figures and results presented in the manuscript been provided?**
Large-scale datasets should be made available via a public repository as described in the *PLOS Computational Biology* [data availability policy](https://urldefense.com/v3/__http:/journals.plos.org/ploscompbiol/s/data-availability__;!!OaZRxjSJGsACmqMyc8s!eVoXBGJM7ZXCg3gU50BWVqwJoKqbwpghUpZTPJIGLWX-0syJLJ5phyIntfIx6CnvfsHq9g$), and numerical data that underlies graphs or summary statistics should be provided in spreadsheet form as supporting information.

Reviewer #1: Yes

Reviewer #2: None

Reviewer #3: Yes

Reviewer #4: Yes

PLOS authors have the option to publish the peer review history of their article ([what does this mean?](https://urldefense.com/v3/__https:/journals.plos.org/ploscompbiol/s/editorial-and-peer-review-process*loc-peer-review-history__;Iw!!OaZRxjSJGsACmqMyc8s!eVoXBGJM7ZXCg3gU50BWVqwJoKqbwpghUpZTPJIGLWX-0syJLJ5phyIntfIx6CmqnZEj5A$)). If published, this will include your full peer review and any attached files.


If you choose “no”, your identity will remain anonymous but your review may still be made public.


**Do you want your identity to be public for this peer review?** For information about this choice, including consent withdrawal, please see our [Privacy Policy](https://urldefense.com/v3/__https:/www.plos.org/privacy-policy__;!!OaZRxjSJGsACmqMyc8s!eVoXBGJM7ZXCg3gU50BWVqwJoKqbwpghUpZTPJIGLWX-0syJLJ5phyIntfIx6CnPbFbheQ$).

Reviewer #1: No

Reviewer #2: **Yes:** Veit Zoche-Golob

Reviewer #3: **Yes:** A K Chattopadhyay

Reviewer #4: No

Figure Files:

While revising your submission, please upload your figure files to the Preflight Analysis and Conversion Engine (PACE) digital diagnostic tool, [https://pacev2.apexcovantage.com](https://urldefense.com/v3/__https:/pacev2.apexcovantage.com/__;!!OaZRxjSJGsACmqMyc8s!eVoXBGJM7ZXCg3gU50BWVqwJoKqbwpghUpZTPJIGLWX-0syJLJ5phyIntfIx6CmjsAagdA$). PACE helps ensure that figures meet PLOS requirements. To use PACE, you must first register as a user. Then, login and navigate to the UPLOAD tab, where you will find detailed instructions on how to use the tool. If you encounter any issues or have any questions when using PACE, please email us at [figures@plos.org](mailto:figures@plos.org).

Data Requirements:

Please note that, as a condition of publication, PLOS' data policy requires that you make available all data used to draw the conclusions outlined in your manuscript. Data must be deposited in an appropriate repository, included within the body of the manuscript, or uploaded as supporting information. This includes all numerical values that were used to generate graphs, histograms etc.. For an example in PLOS Biology see here: [http://www.plosbiology.org/article/info%3Adoi%2F10.1371%2Fjournal.pbio.1001908#s5](https://urldefense.com/v3/__http:/www.plosbiology.org/article/info*3Adoi*2F10.1371*2Fjournal.pbio.1001908*s5__;JSUlIw!!OaZRxjSJGsACmqMyc8s!eVoXBGJM7ZXCg3gU50BWVqwJoKqbwpghUpZTPJIGLWX-0syJLJ5phyIntfIx6CnmOetRcQ$).

Reproducibility:

To enhance the reproducibility of your results, we recommend that you deposit your laboratory protocols in protocols.io, where a protocol can be assigned its own identifier (DOI) such that it can be cited independently in the future. Additionally, PLOS ONE offers an option to publish peer-reviewed clinical study protocols. Read more information on sharing protocols at [https://plos.org/protocols?utm_medium=editorial-email&utm_source=authorletters&utm_campaign=protocols](https://urldefense.com/v3/__https:/plos.org/protocols?utm_medium=editorial-email&utm_source=authorletters&utm_campaign=protocols__;!!OaZRxjSJGsACmqMyc8s!eVoXBGJM7ZXCg3gU50BWVqwJoKqbwpghUpZTPJIGLWX-0syJLJ5phyIntfIx6CkAqtpDdw$)
