# Supplementary material for: Building and experimenting with an agent-based model to study the population-level impact of CommunityRx, a clinic-based community resource referral intervention
Source: PLoS Comput Biol. 2021 Oct 25;17(10):e1009471. doi: 10.1371/journal.pcbi.1009471 (PMC8568099; doi:10.1371/journal.pcbi.1009471)
Supplement: Attachment — Submitted filename: Response.docx [file pcbi.1009471.s004.docx]

**The authors have responded to all the issues raised as shown below, therefore, it is recommended for the paper to be accepted.**

**Reviewer #4:** The following are my general comments of the article

The article shows a coherent story of the study, the authors have taken the time to explicitly explained the whole concept of Agent Based Modeling and its associated procedures. The arrangements were made in such a way that, it predominantly centered on language understandable by all thus non-technical interpretations were offered.

In as much as the reviewer believes detailed procedures and well explained methodology and implementation understandable by ordinary people with interest in science, the author also suggests the following below for the authors to reconsider in providing further explanations to make the concepts much clearer.

Report on Reviewed article

COMMENT:

Line 133 The authors should consider deleting line 133 sentence as it doesn’t add up to the sentence. It can be placed at where funding information is required to be place 133 With initial funding from a Center for Medicare and Medicaid Innovation award (3/2013- 5/2015, 134 “CRx-1”),

Authors: We have made this edit.

COMMENT: Line 135 I believe the authors were referring to tens of thousands instead of "thens" of thousands 135 30 month period. All patients living in the target geography were eligible, including thens of 136 thousands of people with cancer, cardiovascular disease, pulmonary disease, obesity and other

Authors: The reviewer is correct. We have made this edit.

COMMENT: Line 137 Authors should include the unit of measurement of age, “18-93 years”

137 common chronic conditions. In a telephone survey of 458 HealtheRx recipients ages 18-93,

Authors: We have made this edit.

COMMENT:

Line 197 This statement should be appropriately recapture and the link should be inserted as part of the appendix. Moreover, authors should clearly show which part is in a bracket and which is not, the end of the link has a close bracket but the beginning of the bracket is not

seen.

197 toolkits. The model code and the workflow code used to implement the parameter space

198 characterization experiments are publicly available at https://github.com/jozik/communityrx).

Authors: We have fixed the bracketing in the manuscript.

COMMENT: Line 250. What informed this categorization, any special reasons? as why not other age groups different from what you have chosen?

250 was computed within each of four age groups (<31, 31-45, 46-65 and 65+ years), weighted

by

Authors: We have addressed the age group question with the following statement in the revised Methods Section 3.5.1 “Agent activity behavior”: “These groups were defined using demographic, life course and healthcare policy considerations and are consistent with commonly applied strata for age in the clinical and population health literature.”

COMMENT: Line 276: Can authors throw more light as to how (inherent inertia) this was captured or measured?

As it could be a source of uncertainty in the model itself? Was it also derived from Skolasky et al?

276 iii) the inherent inertia that they needed to overcome for using that service (γj).

Authors: Agent inertia was parameterized into Low, Medium, and High based on the difficulty associated with performing an activity at a resource. The assignment of low, medium, or high was done through expert informant surveys completed by a diversity of researchers with expertise about the geographic region and its population. The parameter values for gamma (inertia) values were set during model calibration runs, ensuring that model output matched empirical visits. Details on the calibration process are available in previously published work (Kaligotla, 2020).

COMMENT: Line 305/308: In equation presentation, variables/parameters, constants are explained just below the equations, in equation 2, since all variables have been previously explained, could authors given meaning to the value, εx, where ∈?. Moreover, εx could have been any value, why the chosen values on line 308?

305: of resource information was associated with a value, εx, where ∈

308: used to produce Figure 5, the following values were used = {0.05, 0.15, 0.25, 0.2, 0.9, 1}.

Authors: The relative values for Figure 5 were informed by expert informants and through sensitivity analyses. The goal was to reproduce the evolutionary dynamics of resource information knowledge at an agent level. Epsilon values for the different dosing sources (x) are set between 0 and 1 to ensure that the Beta score remains between 0 and

1. Indeed, a range of values for each epsilon(x) is possible, with each parameter value dictating the relative weights for sources of information. As a modeling choice, we set the

relative epsilon values for the different sources, i.e., dosing from a doctor is weighted

higher (lower epsilon score) than dosing from a peer (higher epsilon score). Values for

some of these parameters (dosing decay and peer dosing) were set during the calibration

process described in Kaligotla, 2020

NOTE (Not necessarily a required consideration, but a simple explanation could help, on below

general comments)

1. It is widespread believed that, the alternative version of ABM could be made in differential

equations since its well suited in dynamical systems of how agents behave (though the debate

has not settled yet within the mathematical science fraternity), could the authors consider explaining why ABM is well suited for this study but not any of the Complex Adaptive Systems

Modeling techniques (Complex Network Modeling Level, Exploratory Agent-based Modeling

Level, Validated agent-based modeling and Descriptive Agent-based Modeling )?

Authors: An ABM approach allows us to model behaviors at an individual agent level while considering heterogeneous agents, something that becomes exceptionally challenging while using the differential equations approach. Furthermore, an ABM approach allows us to model heterogeneous agent behavior to result in endogenous colocation networks (that indicate information pathways). This approach enables us to tie in individual choice behavior to emergent population-level outcomes (like total visits to a clinic or other health maintenance activities performed). The micro-to-macro level

dynamics make ABMs particularly well suited to study an informational intervention.

2. The GitHub link provided by the author: Authors should consider adding in-depth comments to the scripts provided in the GitHub in order for users who might "clone the scripts" be able to understand what the lines of codes were meant for, for the sake of reproducibility of work which is the cornerstone of today's scientific study dissemination.

Authors: We agree with the reviewer and have provided additional references and guidance in the Github repository.
